# Management of severe ME/CFS in children and young people in the UK: a British Paediatric Surveillance Unit study

**DOI:** 10.1136/bmjpo-2023-002436

**Published:** 2024-03-07

**Authors:** Alexander Peter Royston, Sarah Burge, Ilaria Idini, Amberly Brigden, Katharine Claire Pike

**Affiliations:** 1 Centre for Academic Child Health, University of Bristol Medical School, Bristol, UK; 2 University Hospitals Bristol and Weston NHS Foundation Trust, Bristol, UK; 3 Royal United Hospitals Bath NHS Foundation Trust, Bath, UK; 4 School of Engineering Mathematics and Technology, University of Bristol, Bristol, UK

**Keywords:** Chronic Fatigue Syndrome, Epidemiology, Adolescent Health

## Abstract

**Objective:**

Severe myalgic encephalomyelitis or chronic fatigue syndrome (ME/CFS) in children and young people (CYP) is a little-understood condition which significantly impacts education, development and quality of life. We used data from a population-wide surveillance study to explore the screening investigation, referral and management of suspected cases of paediatric severe ME/CFS.

**Methods:**

A British Paediatric Surveillance Unit (BPSU) study reported cases of CYP with suspected severe ME/CFS between February 2018 and February 2019. Paediatricians reporting cases to BPSU and allied healthcare professionals in two large specialist paediatric ME/CFS centres were invited to complete questionnaires for CYP meeting the surveillance case definition. The study focused primarily on CYP with confirmed severe ME/CFS and the extent to which their care met NICE (The National Institute for Health and Care Excellence) recommendations but also considered separately those with probable or possible severe ME/CFS.

**Results:**

This study includes a total of 92 CYP with suspected severe ME/CFS; 33 meeting criteria for severe ME/CFS and an additional 59 classified as probable or possible severe ME/CFS. For 16 possible cases, incomplete investigation to exclude alternative diagnoses prevented confirmation of a severe ME/CFS diagnosis. Only 21 of 33 (64%) confirmed severe ME/CFS cases had been referred to specialist services. The management provided varied considerably between patients and four received nothing at all. Of the management provided, the most frequent approaches were medication (67%), activity management (61%) and physiotherapy (61%). Domiciliary assessments and support, and social services referrals were received by 12% and 6% of confirmed severe cases. Similar proportions of management approaches were seen in probable/possible severe ME/CFS.

**Conclusion:**

Full investigation is frequently incomplete in CYP with suspected severe ME/CFS and recommendations for referral and management are poorly implemented, in particular the needs of CYP who are unable to leave their home might be poorly met.

**WHAT IS ALREADY KNOWN ON THIS TOPIC**

- Myalgic encephalomyelitis, also known as chronic fatigue syndrome (ME/CFS), is a disabling condition that affects people of all ages, including children and young people (CYP).
- NICE (The National Institute for Health and Care Excellence) published guidelines in 2021 recommending that CYP with suspected severe ME/CFS should have standardised screening investigations to exclude conditions other than ME/CFS that might cause similar symptoms.
- Those with a confirmed diagnosis should be referred to specialist care and this should include domiciliary visits, particularly for individuals unable to leave their homes. It is not known how often these recommendations are implemented.

**WHAT THIS STUDY ADDS**

- The standardised investigations recommended by NICE are inconsistently performed for CYP with suspected severe ME/CFS, and this is a barrier to confirmation of diagnosis.
- Referral guidelines are not met for nearly one-third of CYP diagnosed with severe ME/CFS.
- Medication to manage symptoms is prescribed for the majority but domiciliary visits and social service referrals are quite uncommon, received by only 12% of confirmed cases.

**HOW THIS STUDY MIGHT AFFECT RESEARCH, PRACTICE OR POLICY**

- This study suggests the provision for CYP with ME/CFS, including appropriate investigation and onward referral to specialist services requires standardisation in clinical practice.
- Further research is required to identify barriers to the NICE guideline’s implementation and how these can be tackled.

## Introduction

Myalgic encephalomyelitis/chronic fatigue syndrome (ME/CFS) is a disabling illness of unclear aetiology. There are several diagnostic frameworks for ME/CFS^1–4^; each describes symptoms including fatigue, postexertional malaise,^5^ muscle and joint pain, disturbed sleeping^5^ and difficulties concentrating.^6^ Severely affected individuals can have limited mobility, be unable to leave their home and have severe cognitive difficulties. ME/CFS can have a major impact on children and young people’s (CYP) development and functioning. Reduced school attendance is a particularly significant problem,^7^ impacting development, education and career plans.^8–10^

As recently as the 1990s, the prevalence of ME/CFS in teenagers was unknown and there was little acknowledgement that primary school-age children can be affected.^11^ A recent surveillance study conducted in the United Kingdom (UK) and the Republic of Ireland (RoI) reported 33 confirmed severe ME/CFS cases in CYP between 5 and 16 years of age, suggesting a prevalence of 3.18 per million children (95% CI 2.19 to 4.47).^12^ However, epidemiological prevalence estimates vary depending on the diagnostic criteria and methodology used and on whether the thorough clinical investigation has been completed to confirm a diagnosis. The 2021 NICE guidelines include recommendations about screening investigations necessary to exclude alternative causes of fatigue.^13–16^

NICE (The National Institute for Health and Care Excellence) recommends referral to a specialist centre in order to confirm diagnosis and develop a care and support plan. Since CYP with severe ME/CFS can be unable to leave their home, it can be difficult for them to attend outpatient clinics and in the absence of outreach services their health needs may not be met.^17 18^ NICE guidelines recommend home visits to conduct holistic assessments to enable the development of care and support plans by a ME/CFS specialist team.^6^

A scoping study conducted of UK services treating adults with severe ME/CFS reported that 55% (27 of 49) met NICE guidelines for treatment of severe ME/CFS and 12% (6 of 49) offered occasional support where funding allowed. However, 33% (16 of 49) of ME/CFS services provided no service for housebound patients.^19^ No equivalent study has been conducted in CYP and it is currently difficult to evaluate or plan service provision for this population.

We aimed to explore whether the condition is fully investigated in the UK and RoI (as per NICE guidelines), whether appropriate referrals are made to specialist services and the range of managements delivered.

## Method

### Study design

Data were collected using a prospective cross-sectional questionnaire. Using the British Paediatric Surveillance Unit’s (BPSU)^20^ reporting system for rare conditions,^12^ between February 2018 and February 2019 paediatricians in the UK and RoI were invited to report CYP aged 5–16 years, meeting a clinical diagnosis of ME/CFS who due to fatigue were able to attend school for an hour or less per week over the last six term-time weeks. Paediatricians reporting cases were asked to complete detailed questionnaires on the cases that they reported. Along with patient demographics, symptomology and function, the questionnaire asked whether the NICE-recommended screening investigations were completed, whether the CYP had been referred to a specialist centre and about the management they received. This study also employed parallel reporting from two large specialist paediatric ME/CFS centres (the Royal United Hospitals Bath NHS Foundation Trust (RUH) and University College London Hospital (UCLH)). In these centres, CYP are assessed and treated by allied healthcare professionals who do not receive BPSU survey requests. Questionnaires were distributed to professionals working in the RUH and UCLH ME/CFS centres to complete for CYP under management at that centre meeting the surveillance case definition.

### Case definition

A staged process was used to confirm severe ME/CFS; this required screening blood tests to exclude alternative diagnoses and assessment of symptom characteristics, duration and impact.^12^ Patients with abnormal blood tests suggestive of another cause for the fatigue were classified as not severe ME/CFS and excluded from further analysis. Urinalysis is recommended within the NICE guideline, but this was removed as a mandatory criterion by the study team because it was so often missed. Patients otherwise meeting the case definition but for whom it was unclear whether they had experienced a significant reduction in activities of daily living were classified as having probable severe ME/CFS. Those meeting most criteria but with incomplete screening tests or information on function were classified as having possible severe ME/CFS.

In the absence of an official paediatric ME/CFS specialist service specification, a list of specialist services was compiled from services containing a paediatrician and treating CYP that were named either in a list published by Great Ormond Street Hospital for Children^21^ or the 2018 directory^22^ of the British Association of Clinicians in ME/CFS. The questionnaire asked whether the CYP had received any of the following treatments/assessments: activity management, cognitive–behavioural therapy (CBT), domiciliary assessment or treatment, graded exercise therapy, medication for symptoms, physiotherapy, social services assessment and support, or treatment from Child and Adolescent Mental Health Services (CAMHS). These approaches were in line with NICE guidelines at the time of study design,^23^ though more recent guidelines also recommend dietetic assessments for certain CYP and prohibit graded exercise therapy.^6^

### Data analysis

Data were analysed using Stata V.16.0 (StataCorp, College Station, Texas, USA) and expressed using appropriate summary statistics. The primary analysis focused on identifying the proportion of CYP with confirmed severe ME/CFS that were appropriately referred to specialist services and reporting the treatments offered to this group. Secondary analyses reported (1) demographics, referral and management of children with probable or possible severe ME/CFS and (2) screening investigations completed in CYP with possible severe ME/CFS.

### Patient and public involvement

The study idea and its proposal were developed with Action for ME and the CFS/ME Patient Advisory Group (PAG) was both involved in the study design and writing public information leaflets.

## Results

Questionnaires were completed for 92 CYP with a clinical diagnosis of ME/CFS significantly impacting school attendance. Sixty-four cases were identified from the BPSU surveillance survey and 28 from the RUH and UCLH specialist centres. Severe ME/CFS was confirmed for 33 CYP. The functional criterion was not met, or functional status or investigation data were missing in 59 cases, which were defined as probable or possible severe ME/CFS. Nineteen of 33 confirmed severe cases (58%) were female, 32 (97%) were white and the median age of fatigue onset was 13.0 years (IQR 11.4–14.5) (table 1). Demographic characteristics were similar in the possible/probable cases (table 1).

**Table 1:** Demographics and investigations for CYP with severe and possible/probable ME/CFS

Characteristics	Severe ME/CFS		Possible/probable ME/CFS		
		N=33		N	
Female, n (%)	19	(58)	31	58	(54)
Ethnicity A1, n (%)	32	(97)	54	56	(96)
Age at diagnosis, median (IQR)	13.0	(11.4–14.5)	14.3	50	(13.1–15.4)
Reporting method					
BPSU, n (%)	21	(64)	43	59	(73)
Directly reported from specialist centre	12	(36)	16	59	(27)
Investigations					
Patients with all diagnostic investigations complete, n (%)	33	(100)	35	59	(59)
Patients with all investigations required for management complete, n (%)	33	(100)	41	59	(70)

BPSU, British Paediatric Surveillance Unit; CYP, children and young people; ME/CFS, myalgic encephalomyelitis or chronic fatigue syndrome.

### Screening investigations

To meet the case definition of confirmed severe ME/CFS, all investigations were complete and reported as normal in confirmed severe ME/CFS cases (except for urinalysis in 11 cases, (33%)) (table 1). Of the possible/probable cases, only 15 cases had complete investigations. The proportion reporting a blood result varied by test. Urea, electrolytes and creatinine, and full blood count were the most commonly reported (86%), while random blood glucose and creatine kinase were less reported (64% and 67%, respectively). Urinalysis was completed and reported as normal in 14 CYP (26%).

### Referral

Only 64% (21/33) of patients with confirmed severe ME/CFS had been referred to a specialist paediatric centre, 11 (33%) after referral from a non-specialist paediatrician and 10 (30%) after referral from primary care (table 2). Of the 12 CYP with confirmed severe ME/CFS not referred to a specialist centre, three were referred between centres classified in this study as non-specialist.

**Table 2:** Reporting and referrals of CYP with severe and possible/probable ME/CFS

Reporting and referrals	Severe ME/CFS N=33	Possible/probable ME/CFS N=59
Referrals		
From paediatrician to specialist centre, n (%)	11 (33)	30 (51)
Seen by specialist centre directly, n (%)	10 (30)	8 (14)
Total: seen by specialist services	21 (64)	38 (64)
Referred between paediatric services (non-specialist), n (%)	3 (9)	3 (5)
Not referred	9 (27)	18 (31)
Total: not seen in specialist services	12 (36)	21 (36)

CYP, children and young people; ME/CFS, myalgic encephalomyelitis or chronic fatigue syndrome.

In possible/probable cases, 30 (51%) were referred from a paediatrician to a specialist centre and 8 (14%) were seen by specialist centres directly following referral from primary care. This left 21 possible/probable cases (36%) reported by non-specialists and not referred to a specialist centre, 3 (6%) of whom were referred between non-specialist centres (table 2).

### Management

Among CYP with confirmed severe ME/CFS, 27 had received between one and six management approaches (median 3, IQR 1–4) but 4/33 received no management of any kind (table 3). The most common intervention was medication (22/33: 67%). Few specific examples of medication were given, though melatonin, laxatives and thyroxine were described in free-text responses. Activity management and physiotherapy were the second most used approaches (both used in 61%), while fewer CYP received treatment from CAMHS or CBT (33% and 24%, respectively). Only 4/33 (12%) of severe cases had received a domiciliary visit and 2/33 (6%) had received a social service assessment or support.

**Table 3:** Management received by CYP with confirmed severe, probable and possible severe ME/CFS, showing those managed in specialist services

Management/treatments	Severe ME/CFS			Possible/probable ME/CFS		
	Total	Spec	Non-spec	Total	Spec	Non-spec
	N=33	N=21	N=12	N=59	N=38	N=12
Activity management, n (%)	20 (61)	13 (62)	7 (58)	41 (69)	25 (66)	16 (76)
Cognitive–behavioural therapy, n (%)	8 (24)	6 (29)	2 (17)	10 (17)	9 (24)	1 (5)
Domiciliary assessment/treatment, n (%)	4 (12)	3 (14)	1 (8)	6 (10)	6 (16)	0 (0)
Graded exercise therapy, n (%)	11 (33)	7 (33)	4 (33)	18 (31)	13 (34)	5 (24)
Medication for symptoms, n (%)	22 (67)	14 (67)	8 (67)	30 (51)	20 (53)	10 (48)
Physiotherapy, n (%)	20 (61)	12 (57)	8 (67)	35 (59)	24 (63)	11 (52)
Social services assessment/support, n (%)	2 (6)	2 (10)	0 (0)	10 (17)	6 (16)	4 (19)
Treatment from CAMHS, n (%)	11 (33)	6 (29)	5 (42)	20 (34)	16 (42)	4 (19)
Total number of treatments per patient, n (%)						
0	4 (12)	3 (14)	1 (8)	6 (10)	3 (8)	3 (25)
1–2	9 (27)	5 (24)	4 (33)	20 (34)	12 (32)	9 (75)
3–4	13 (39)	7 (33)	6 (50)	20 (34)	14 (37)	7 (58)
5–6	7 (21)	5 (24)	2 (17)	9 (15)	9 (24)	2 (17)
7–8	0 (0)	0 (0)	0 (0)	0 (0)	0 (0)	0 (0)
Median (IQR)	3.0 (1.0–4.0)			3.0 (2.0–4.0)		

CAMHS, Child and Adolescent Mental Health Services; CYP, children and young people; ME/CFS, myalgic encephalomyelitis or chronic fatigue syndrome.

Forty-nine (83%) CYP with possible/probable severe ME/CFS received between one and six management approaches (median 3, IQR 2–4), while six (10%) received no management (table 3). The proportion receiving physiotherapy was similar in the possible/probable group to that in those with a confirmed diagnosis (59%), though a slightly lower proportion received medication (30/59, 51%) and a higher proportion received activity management (69%). The proportion of children in the probable/possible group who received CAMHS and CBT was similar to that seen in CYP with a confirmed diagnosis. Small numbers received either domiciliary assessment or treatment or social services assessment or support (6/59 (10%) and 10/59 (17%), respectively).

A lower proportion of CYP with confirmed or possible/probable severe ME/CFS seen in specialist centres received activity management or physiotherapy compared with CYP seen in non-specialist centres. The proportion of patients offered specific management approaches appeared similar for those with confirmed or possible/probable severe ME/CFS (table 3). The proportion receiving domiciliary assessment/treatment or social services support was not similar between patients referred or not referred to specialist centres (14% vs 8% (confirmed) and 16% vs 0% (possible/probable)), although numbers are too small to draw definite conclusions from this.

## Discussion

This study has found that the majority of 92 CYP with suspected severe ME/CFS identified via a UK and RoI surveillance study and survey of two large specialist centres did not receive the screening investigations recommended within current NICE guidelines. Severe ME/CFS was confirmed in 33 (36%) cases but up to a further 16 cases might have been confirmed had blood investigations been completed and reported normal. A substantial number of confirmed severe cases of ME/CFS, 12/33 (36%), and similar proportions of probable or possible cases, were not referred to a specialist centre. The median number of management approaches received was three; and while medication (67%), activity management (61%) and physiotherapy (61%) were commonly reported, fewer patients received treatment from CAMHS 11/33 (33%) or CBT 8/33 (24%), and domiciliary assessments and social services support were received by only 12% and 6% of confirmed severe ME/CFS cases.

### Strengths and limitations

The principal strength of this study is that it is a prospective surveillance study using the BPSU’s well-established methodology, which generally achieves a good coverage of paediatricians with response rates in the range of 90%. However, potential limitations arose from the challenges of completing a surveillance study during the COVID-19 pandemic, difficulties were encountered in contacting clinicians and in encouraging questionnaire completion and return. While cases identified by the surveillance study overlapped with those identified from the RUH and UCLH centres, the number of duplicates was low which might suggest case identification was incomplete. Our method potentially underestimates the prevalence of confirmed severe ME/CFS compared with other population-based studies due to the strict case definition criteria used. However, investigation, referral and management characteristics were reported relating to possible and probable cases which might be included under alternative case definitions. Since a high proportion of patients with severe ME/CFS are housebound, making them ‘hard to reach’ and potentially underdiagnosed with poor access to support, this might limit the generalisability of this study’s findings across the CYP severe ME/CFS population.^24^

### Comparison with previous studies

To our knowledge, this is the first national study carried out in children. A national scoping exercise of UK adults^19^ found that while 55% of specialist adult ME/CFS centres met NICE guidelines for service provision for patients with severe ME/CFS, but 33% (16/49) provided no service for housebound patients. The most offered management modalities were activity management, CBT and graded activity. The adult study focused on service provision at the level of specialist centres rather than services provided to individual patients meeting severe ME/CFS criteria.

Although NICE has made recommendations, the evidence for specific management approaches in CYP with severe ME/CFS is scant. While many studies have explored the management of ME/CFS in paediatric populations,^25^ for example, evaluations of exercise therapy^26 27^ and CBT,^28 29^ none have focused on the severe form of the condition. Inclusion criteria typically either broaden the study samples to include those with milder disease (for example, choosing a lower cut-off SF-36 score of 60 for inclusion^30^) or exclude those with severe fatigue. 31 Generally, trials of approaches that have shown some promise in treating ME/CFS report only limited evidence of efficacy in the most severely ill adult patients^32^ and research is lacking in patients of all ages.

### Implications

The finding that a substantial number of CYP with suspected severe ME/CFS have incomplete screening investigations not only affects the accuracy of prevalence estimates but also has diagnostic implications. Treatable conditions that resemble ME/CFS might not be identified, and this can lead to inappropriate management. This potential for harm is recognised in the NICE guidance and underlies the recommendation that prescribing should be initiated under the supervision of a paediatrician with expertise in ME/CFS.^6^ This is a particular concern given that a sizeable proportion of CYP are either without a confirmed diagnosis or under management in non-specialist centres. NICE’s 2021 guidance for ME/CFS of all grades of severity, including those with severe forms of the condition,^6^ recommends clinicians “refer children and young people who have been diagnosed with ME/CFS after assessment by a paediatrician directly to a paediatric ME/CFS specialist team”.

Our results show that only two-thirds of patients with severe ME/CFS were under specialist management when receiving physiotherapy and activity management. NICE recommends specialist physiotherapy advice and additional care for those with severe or very severe ME/CFS when an approach involving energy management has been chosen.^6^ While it is encouraging that CYP with severe ME/CFS have access to physiotherapy, it is possible that they may not be receiving optimal care. It is not known why specialist centres appear to recommend activity management and physiotherapy less often.

The scarcity of domiciliary visits is of concern for this patient group.^33^ The proportion of patients receiving home assessments (12%) falls short of NICE’s recommendations that home visits should be offered to all with severe or very severe ME/CFS.^6^ Social services assessment and support was similarly rare in our cohort (6%), raising the possibility that CYP and their parents or carers are not receiving the support that they are entitled to.

## Conclusion

Investigation, referral and management of CYP with suspected severe ME/CFS do not always meet NICE recommendations. This suggests patients may be receiving suboptimal care since alternative diagnoses are not appropriately excluded and specialist care is not provided. The low number of CYP receiving domiciliary visits or social services assessment/support limits their access to management. Further work should focus not only on determining the most effective treatments for children with severe ME/CFS but also on exploring the barriers to accessing these services and how these can be addressed.

## Supplementary Material

Reviewer comments

Author's manuscript

## Data Availability

No data are available.
