# Supplementary material for: Management of severe ME/CFS in children and young people in the UK: a British Paediatric Surveillance Unit study
Source: BMJ Paediatr Open. 2024 Mar 7;8(1):e002436. doi: 10.1136/bmjpo-2023-002436 (PMC10921487; doi:10.1136/bmjpo-2023-002436)
Supplement: Author's manuscript [file bmjpo-2023-002436.draft_revisions.pdf]

BMJ Paediatrics Open

BMJ Paediatrics Open is committed to open peer review. As part of this commitment we make the peer review history of every article we publish publicly available.

When an article is published we post the peer reviewers' comments and the authors' responses online. We also post the versions of the paper that were used during peer review. These are the versions that the peer review comments apply to.

The versions of the paper that follow are the versions that were submitted during the peer review process. They are not the versions of record or the final published versions. They should not be cited or distributed as the published version of this manuscript.

BMJ Paediatrics Open is an open access journal and the full, final, typeset and author-corrected version of record of the manuscript is available on our site with no access controls, subscription charges or pay-per-view fees (http://bmjpaedsopen.bmj.com).

If you have any questions on BMJ Paediatrics Open's open peer review process please email info.bmjpo@bmj.com

BMJ Paediatrics Open

Management of Severe ME/CFS in Children and Young People in the UK: a British Paediatric Surveillance Unit study

Journal:	*BMJ Paediatrics Open*
Manuscript ID	bmjpo-2023-002436
Article Type:	Original research
Date Submitted by the Author:	06-Dec-2023

Complete List of Authors:	Royston, Alexander; University of Bristol Medical School, Centre for Academic Child Health; University Hospitals Bristol and Weston NHS Foundation Trust<br>Burge, Sarah; University of Bristol Medical School, Centre for Academic Child Health<br>Idini, Ilaria; Royal United Hospitals Bath NHS Foundation Trust<br>Brigden, Amberly; University of Bristol, School of Engineering Mathematics and Technology<br>Pike, Katharine; University of Bristol Medical School, Centre for Academic Child Health

Keywords:	Chronic Fatigue Syndrome, Epidemiology, Adolescent Health

SCHOLARONE™ Manuscripts

I, the Submitting Author has the right to grant and does grant on behalf of all authors of the Work (as defined in the below author licence), an exclusive licence and/or a non-exclusive licence for contributions from authors who are: i) UK Crown employees; ii) where BMJ has agreed a CC-BY licence shall apply, and/or iii) in accordance with the terms applicable for US Federal Government officers or employees acting as part of their official duties; on a worldwide, perpetual, irrevocable, royalty-free basis to BMJ Publishing Group Ltd ("BMJ") its licensees and where the relevant Journal is co-owned by BMJ to the co-owners of the Journal, to publish the Work in this journal and any other BMJ products and to exploit all rights, as set out in our licence.

The Submitting Author accepts and understands that any supply made under these terms is made by BMJ to the Submitting Author unless you are acting as an employee on behalf of your employer or a postgraduate student of an affiliated institution which is paying any applicable article publishing charge ("APC") for Open Access articles. Where the Submitting Author wishes to make the Work available on an Open Access basis (and intends to pay the relevant APC), the terms of reuse of such Open Access shall be governed by a Creative Commons licence – details of these licences and which Creative Commons licence will apply to this Work are set out in our licence referred to above.

Other than as permitted in any relevant BMJ Author's Self Archiving Policies, I confirm this Work has not been accepted for publication elsewhere, is not being considered for publication elsewhere and does not duplicate material already published. I confirm all authors consent to publication of this Work and authorise the granting of this licence.

Confidential: For Review Only

Management of Severe ME/CFS in Children and Young People in the UK: a British Paediatric Surveillance Unit study

Authors: Royston, A[1]., Burge, S[1]., Idini, I[2]., Brigden, A[3]., Pike, K[1]

Journal

1) BMJ Paediatrics Open

Author affiliations

[1] Centre for Academic Child Health, Bristol Medical School, Canynge Hall, 39 Whatley Road, Bristol BS8 2PS, UK

[2] Royal United Hospitals Bath NHS Foundation Trust, Combe Park, Bath BA1 3NG, UK

[3] School of Engineering Mathematics and Technology, University of Bristol, Bristol, BS8 1UB, UK

Corresponding author:

Dr Alexander Royston

ar16623@bristol.ac.uk

Key words

Paediatric, ME/CFS, chronic fatigue syndrome

Confidential: For Review Only

ABSTRACT

Objective: Severe myalgic encephalomyelitis or chronic fatigue syndrome (ME/CFS) in children and young people (CYP) is a little understood condition which significantly impacts education, development and quality of life. We used data from a population-wide surveillance study to explore the screening investigation, referral and management of suspected cases of paediatric severe ME/CFS.

Methods: A British Paediatric Surveillance Unit (BPSU) study reported cases of CYP with suspected severe ME/CFS between February 2018 and February 2019. Paediatricians reporting cases to BPSU and allied healthcare professionals in two large specialist paediatric ME/CFS centres were invited to complete questionnaires for CYP meeting the surveillance case definition. The study focussed primarily on CYP with confirmed severe ME/CFS and the extent to which their care met NICE recommendations, but also considered separately those with probable or possible severe ME/CFS.

Results: This study incudes a total of 92 CYP with suspected severe ME/CFS; 33 meeting criteria for severe ME/CFS and an additional 59 classified as probable or possible severe ME/CFS. For 16 possible cases incomplete investigation to exclude alternative diagnoses prevented confirmation of a severe ME/CFS diagnosis. Only 21 of 33 (64%) confirmed severe ME/CFS cases had been referred to specialist services. The management provided varied considerably between patients and four received nothing at all. Of the management provided, the most frequent approaches were medication (67%), activity management (61%) and physiotherapy (61%). Domiciliary assessments and support, and social services referrals were received by 12% and 6% of confirmed severe cases. Similar proportions of management approaches were seen in probable/possible severe ME/CFS.

Conclusions: Full investigation is frequently incomplete in CYP with suspected severe ME/CFS and recommendations for referral and management are poorly implemented, in particular the needs of CYP who are unable to leave their home might be poorly met.

Confidential: For Review Only

SUMMARY BOX

A funding statement:

The study was funded by The National Institute for Health Research (Senior Research Fellowship, SRF-2013-06-013) and a grant from the Royal United Hospitals Bath NHS Foundation Trust.

Disclosure statement: The authors have declared no conflicts of interests

Any checklist/flow diagram for the appropriate reporting statement: STROBE

Patient consent form: not included – please see CAG Section 251 approval statement in Methods

Please provide a data sharing statement: Data unavailable for review due to being covered by CAG Section 251 approval **(18/CAG/0052)**

Confidential: For Review Only

Key Messages

What is already known on this topic:

Myalgic Encephalomyelitis, also known as Chronic Fatigue Syndrome (ME/CFS), is a disabling condition that affects people of all ages, including children and young people (CYP). NICE published guidelines in 2021 recommending that CYP with suspected severe ME/CFS should have standardised screening investigations to exclude conditions other than ME/CFS that might cause similar symptoms. Those with a confirmed diagnosis should be referred to specialist care and this should include domiciliary visits, particularly for individuals unable to leave their homes.(1) It is not known how often these recommendations are implemented.

What this study adds

The standardised investigations recommended by NICE are inconsistently performed for CYP with suspected severe ME/CFS, and this is a barrier to confirmation of diagnosis. Moreover, referral guidelines are not met for nearly one third of CYP diagnosed with severe ME/CFS. Medication to manage symptoms are prescribed for the majority but domiciliary visits and social service referrals are quite uncommon, received by only 12% of confirmed cases.

How this study might affect research, practice or policy

This study suggests the provision for CYP with ME/CFS, including appropriate investigation and onward referral to specialist services requires standardisation in clinical practise. Further research is required to identify barriers to the NICE guideline's implementation and how these can be tackled.

Confidential: For Review Only

INTRODUCTION

Myalgic Encephalomyelitis/Chronic Fatigue Syndrome (ME/CFS) is a disabling illness of unclear aetiology. There are several diagnostic frameworks for ME/CFS(1-4); each describes symptoms including fatigue, post exertional malaise (PEM)(5), muscle and joint pain, disturbed sleeping(5), and difficulties concentrating(6). Severely affected individuals can have limited mobility, be unable to leave their home and have severe cognitive difficulties. ME/CFS can have a major impact on children and young people's (CYP) development and functioning. Reduced school attendance is a particularly significant problem(7), impacting on development, education and career plans(8-10).

As recently as the 1990s, the prevalence of ME/CFS in teenagers was unknown and there was little acknowledgement that primary school-age children can be affected(11). A recent surveillance study conducted in the United Kingdom (UK) and Republic of Ireland (RoI) reported 33 confirmed severe ME/CFS cases in CYP between 5 and 16 years of age, suggesting a prevalence of 3.18 per million children (95%CI 2.19 to 4.47)(12). However, epidemiological prevalence estimates vary depending upon the diagnostic criteria and methodology used and upon whether thorough clinical investigation has been completed to confirm a diagnosis. The 2021 NICE guidelines include recommendations about screening investigations necessary to exclude alternative causes of fatigue(13-16).

NICE recommends referral to a specialist centre in order to confirm diagnosis and develop a care and support plan. Since CYP with severe ME/CFS can be unable to leave their home, it can be difficult for them to attend outpatient clinics and in the absence of outreach services their health needs may not be met(17,18). NICE guidelines recommend home visits to conduct holistic assessments to enable the development of care and support plans by a ME/CFS specialist team(6).

A scoping study conducted of UK services treating adults with severe ME/CFS reported that 55% (27 of 49) met NICE guidelines for treatment of severe ME/CFS and 12% (6 of 49)

Confidential: For Review Only

offered occasional support where funding allowed. However, 33% (16 of 49) of ME/CFS services provided no service for housebound patients(19). No equivalent study has been conducted in CYP and it is currently difficult to evaluate or plan service provision for this population.

We aimed to explore whether the condition is fully investigated in the UK and Republic Ireland (as per NICE guidelines), whether appropriate referrals are made to specialist services and the range of managements delivered.

Confidential: For Review Only

METHOD

Study Design

Data was collected using a prospective cross-sectional questionnaire. Using the BPSU's(20) reporting system for rare conditions(12), between February 2018 and February 2019 paediatricians in the UK and Republic of Ireland were invited to report 5 - 16-year-old CYP, meeting a clinical diagnosis of ME/CFS who due to fatigue were able to attend school for an hour or less per week over the last six term-time weeks. Paediatricians reporting cases were asked to complete detailed questionnaires on the cases that they reported (Appendix 1). Along with patient demographics, symptomology and function, the questionnaire asked whether the NICE-recommended screening investigations were completed, whether the CYP had been referred to a specialist centre, and about the management they received. This study also employed parallel reporting from two large specialist paediatric ME/CFS centres (the Royal United Hospitals Bath NHS Foundation Trust and University College London Hospital, London). In these centres CYP are assessed and treated by allied healthcare professionals who do not receive BPSU survey requests. Questionnaires were distributed to professionals working in the RUH and UCLH ME/CFS centres to complete for CYP under management at that centre meeting the surveillance case definition.

Case Definition

A staged process was used to confirm severe ME/CFS; this required screening blood tests to exclude alternative diagnoses, and assessment of symptom characteristics, duration and impact(12). Patients with abnormal blood tests suggestive of another cause for the fatigue were classified as not severe ME/CFS and excluded from further analysis. Urinalysis is recommended within the NICE guideline, but this was removed as a mandatory criterion by the study team because it was so often missed. Patients otherwise meeting the case definition but for whom it was unclear whether they had experienced a significant reduction in activities of daily living were classified as having probable severe ME/CFS. Those meeting

Confidential: For Review Only

most criteria but with incomplete screening tests or information on function were classified as having possible severe ME/CFS.

In the absence of an official paediatric ME/CFS specialist service specification, a list of specialist services was compiled from services containing a paediatrician and treating CYP that were named either in a list published by Great Ormond Street Hospital for Children(21) or the 2018 directory(22) of the British Association of Clinicians in ME/CFS (BACME). The questionnaire asked whether the CYP had received any of the following treatments/assessments: activity management, cognitive behaviour therapy (CBT), domiciliary assessment or treatment, graded exercise therapy, medication for symptoms, physiotherapy, social services assessment and support, or treatment from Child and Adolescent mental Health Services (CAMHS). These approaches were in line with NICE guidelines at the time of study design(23), though more recent guidelines also recommend dietetic assessments for certain CYP and prohibit graded exercise therapy(6).

Data Analysis

Data were analysed using Stata V16.0 (Statcorp,Tx) and expressed using appropriate summary statistics. The primary analysis focused upon identifying the proportion of CYP with confirmed severe ME/CFS that were appropriately referred to specialist services and reporting the treatments offered to this group. Secondary analyses reported 1) demographics, referral, and management of children with probable or possible severe ME/CFS and 2) screening investigations completed in CYP with possible severe ME/CFS.

Patient and Public Involvement

The study idea and its proposal were developed with Action for ME and the CFS/ME Patient Advisory Group was both involved in the study design and writing public information leaflets.

Ethical approval

Confidential: For Review Only

This study was approved by the BPSU Scientific Committee; National Research Ethics System Committee South-West-Central Bristol Research Ethics Committee (Ref: 18/SW/0051; IRAS ID: 223838); Section 251 Confidentiality Advisory Group permission (Ref: 18/CAG/00051); and Public Benefit and Privacy Panel for Health and Social Care approval (Ref: 1718-0341).

Confidential: For Review Only

RESULTS

Questionnaires were completed for 92 CYP with a clinical diagnosis of ME/CFS significantly impacting school attendance. Sixty-four cases were identified from the BPSU surveillance survey and 28 from the RUH and UCLH specialist centres. Severe ME/CFS was confirmed for 33 CYP. The functional criterion was not met, or functional status or investigation data were missing in 59 cases, which were defined as probable or possible severe ME/CFS. Nineteen of 33 confirmed severe cases (58%) were female, 32 (97%) were white and median age of fatigue onset was 13.0 years [IQR 11.4, 14.5](Table 1). Demographic characteristics were similar in the possible/probable cases(Table 1).

Screening investigations

To meet the case definition of confirmed severe ME/CFS all investigations were complete and reported as normal in confirmed severe ME/CFS cases, (except for urinalysis in 11 cases, (33%))(Table 1). Of the possible/probable cases, only 15 cases had complete investigations. The proportion reporting a blood result varied by test. Urea, electrolytes and creatinine, and full blood count were the most commonly reported (86%), whilst random blood glucose and creatine kinase were less reported (64% and 67%, respectively). Urinalysis was completed and reported as normal in 14 CYP (26%).

Referral

Only 64% (21/33) of patients with confirmed severe ME/CFS had been referred to a specialist paediatric centre, 11 (33%) after referral from a non-specialist paediatrician and 10 (30%) after referral from Primary Care(Table 2). Of the 12 CYP with confirmed severe ME/CFS not referred to a specialist centre, three were referred between centres classified in this study as non-specialist.

Confidential: For Review Only

In possible/probable cases, 30 (51%) were referred from a paediatrician to a specialist centre and 8 (14%) were seen by Specialist centres directly following referral from Primary Care. This left 21 possible/probable cases (36%) reported by non-specialists and not referred to a specialist centre, 3 (6%) of whom were referred between non-specialist centres(Table 2).

Management

Amongst CYP with confirmed severe ME/CFS, 27 had received between one and six management approaches (median 3, IQR 1-4) but 4/33 received no management of any kind(Table 3). The commonest intervention was medication (22/33: 67%). Few specific examples of medication were given, though melatonin, laxatives and thyroxine were described in free-text responses. Activity management and physiotherapy were the second most used approaches (both used in 61%), whilst fewer CYP received treatment from CAMHS or CBT (33% and 24%, respectively). Only 4/33 (12%) of severe cases had received a domiciliary visit and 2/33 (6%) social service assessment or support.

Forty-nine (83%) CYP with possible/probable severe ME/CFS received between one and six management approaches (median 3, IQR 2, 4), whilst six (10%) received no management(Table 3). The proportion receiving physiotherapy was similar in the possible/probable group to that in those with a confirmed diagnosis (59%), though a slightly lower proportion received medication (30/59, 51%) and higher proportion received activity management (69%). The proportion of children in the possible/possible group who received CAMHS and CBT was similar to that seen in CYP with a confirmed diagnosis. Small numbers received either domiciliary assessment or treatment or social services assessment or support (6/59 (10%) and 10/59 (17%), respectively).

Confidential: For Review Only

A lower proportion of CYP with confirmed or possible/probable severe ME/CFS seen in specialist centres received activity management or physiotherapy compared with CYP seen in non-specialist centres. The proportion of patients offered specific management approaches appeared similar for those with confirmed or possible/probable severe ME/CFS(Table 3). The proportion receiving domiciliary assessment/treatment or social services support was not similar between patients referred or not referred to specialist centres (14% vs 8% [confirmed] and 16% vs 0% [possible/probable]), although numbers are too small to draw definite conclusions from this.

Confidential: For Review Only

DISCUSSION

This study has found that the majority of 92 CYP with suspected severe ME/CFS identified via a UK and RoI surveillance study and survey of two large specialist centres did not receive the screening investigations recommended within current NICE guidelines. Severe ME/CFS was confirmed in 33 (36%) cases but up to a further 16 cases might have been confirmed had blood investigations been completed and reported normal. A substantial number of confirmed severe cases of ME/CFS, 12/33 (36%), and similar proportions of probable or possible cases, were not referred to a specialist centre. The median number of management approaches received was three; and whilst medication (67%), activity management (61%) and physiotherapy (61%) were commonly reported, fewer patients received treatment from CAMHS 11/33 (33%) or CBT 8/33 (24%), and domiciliary assessments, and social services support were received by only 12% and 6% of confirmed severe ME/CFS cases.

Strengths and limitations

The principal strength of this study is that it is a prospective surveillance study using the BPSU's well-established methodology, which generally achieves a good coverage of paediatricians with response rates in the range of 90%. However, potential limitations arose from the challenges of completing a surveillance study during the COVID-19 pandemic, difficulties were encountered in contacting clinicians and in encouraging questionnaire completion and return. Whilst cases identified by the surveillance study overlapped with those identified from the RUH and UCLH centres, the number of duplicates was low which might suggest case identification was incomplete. Our method potentially underestimates prevalence of confirmed severe ME/CFS compared to other population-based studies due to the strict case definition criteria used. However, investigation, referral and management characteristics were reported relating to possible and probable cases which might be included under alternative case definitions. Since a high proportion of patients with severe ME/CFS are housebound, making them 'hard to reach' and potentially underdiagnosed with poor access to support, this might limit the generalisability of this study's findings across the CYP severe ME/CFS population(24).

Confidential: For Review Only

Comparison with previous studies

To our knowledge, this is the first national study carried out in children. A national scoping exercise of UK adults(19), found that whilst 55% of specialist adult ME/CFS centres met NICE guidelines for service provision for patients with severe ME/CFS, but 33% (16/49) provided no service for housebound patients. The most offered management modalities were activity management, CBT and graded activity. The adult study focussed on service provision at the level of specialist centres rather than services provided to individual patients meeting severe ME/CFS criteria.

Although NICE has made recommendations, the evidence for specific management approaches in CYP with severe ME/CFS is scant. Whilst many studies have explored management of ME/CFS in paediatric populations(25), for example evaluations of exercise therapy(26,27) and CBT(28,29), none have focused on the severe form of the condition. Inclusion criteria typically either broaden the study samples to include those with milder disease (for example choosing a lower cut-off SF-36 score of 60 for inclusion(30)) or exclude those with severe fatigue ((31)). Generally, trials of approaches that have shown some promise in treating ME/CFS report only limited evidence of efficacy in the most severely ill adult patients(32) and research is lacking in patients of all ages.

Implications

The finding that a substantial number of CYP with suspected severe ME/CFS have incomplete screening investigations, not only affects the accuracy of prevalence estimates but also has diagnostic implications. Treatable conditions that resemble ME/CFS might not be identified, and this can lead to inappropriate management. This potential for harm is recognised in the NICE guidance and underlies the recommendation that prescribing should be initiated under the supervision of a paediatrician with expertise in ME/CFS(6). This is a particular concern given that a sizeable proportion of CYP are either without a confirmed diagnosis or under management in non-specialist centres. NICE's 2021 guidance for ME/CFS of all grades of severity including those with severe forms of the condition(6) and recommends clinicians "refer children and young people who have been diagnosed with ME/CFS after assessment by a paediatrician directly to a paediatric ME/CFS specialist team".

Confidential: For Review Only

Our results show that only two thirds of patients with severe ME/CFS were under specialist management when receiving physiotherapy and activity management. NICE recommends specialist physiotherapy advice and additional care for those with severe or very severe ME/CFS, when an approach involving energy management has been chosen(6). Whilst it is encouraging that CYP with severe ME/CFS have access to physiotherapy, it is possible that they may not be receiving optimal care. It is not known why specialist centres appear to recommend activity management and physiotherapy less often.

The scarcity of domiciliary visits is of concern for this patient-group(33). The proportion of patients receiving home assessments (12%) falls short of NICE's recommendations that home visits should be offered to all with severe or very severe ME/CFS(6). Social services assessment and support was similarly rare in our cohort (6%), raising the possibility that CYP and their parents or carers are not receiving the support that they are entitled to.

Confidential: For Review Only

CONCLUSION

Investigation, referral and management of CYP with suspected severe ME/CFS do not always meet NICE recommendations. This suggests patients may be receiving suboptimal care since alternative diagnoses are not appropriately excluded and specialist care not provided. The low number of CYP receiving domiciliary visits or social services assessment/support limits their access to management. Further work should focus, not only on determining the most effective treatments for children with severe ME/CFS, but also on exploring the barriers to accessing these services and how these can be addressed.

Confidential: For Review Only

Characteristics	Severe ME/CFS *n=33*		Possible/Probable ME/CFS *n*		
Female, n (%)	19	(58)	31	58	(54)
Ethnicity A1, n (%)	32	(97)	54	56	(96)
Age at diagnosis, median (IQR)	13.0	(11.4, 14.5)	14.3	50	(13.1, 15.4)
**Reporting method**					
BPSU, n (%)	21	(64)	43	59	(73)
Directly reported from Specialist centre	12	(36)	16	59	(27)
**Investigations**					
Patients with all diagnostic investigations complete, n (%)	33	(100)	35	59	(59)
Patients with all investigations required for management complete, n (%)	33	(100)	41	59	(70)

Table 1: Demographics and investigations for CYP with severe and possible/probable ME/CFS BACK

Reporting and Referrals	Severe ME/CFS *n=33*		Possible/Probable ME/CFS *n=59*	
**Referrals**				
From paediatrician to Specialist centre, n (%)	11	(33)	30	(51)
Seen by Specialist centre directly, n (%)	10	(30)	8	(14)
**Total:** seen by Specialist services	21	(64)	38	(64)
Referred between paediatric services (non-specialist), n (%)	3	(9)	3	(5)
Not referred	9	(27)	18	(31)
**Total:** not seen in Specialist services	12	(36)	21	(36)

Table 2: Reporting and referrals of CYP with severe and possible/probable ME/CFS BACK

Management/Treatments	Severe ME/CFS			Possible/Probable ME/CFS		
	Total n=33 (%)	*Spec n=21 (%)*	*Non-spec n=12 (%)*	*Total* n=59 (%)	*Spec n=38 (%)*	*Non-spec n=12 (%)*
Activity Management, n (%)	20 (61)	13 (62)	7 (58)	41 (69)	25 (66)	16 (76)
Cognitive Behavioural Therapy, n (%)	8 (24)	6 (29)	2 (17)	10 (17)	9 (24)	1 (5)
Domiciliary assessment/treatment, n (%)	4 (12)	3 (14)	1 (8)	6 (10)	6 (16)	0 (0)
Graded Exercise Therapy, n (%)	11 (33)	7 (33)	4 (33)	18 (31)	13 (34)	5 (24)
Medication for Symptoms, n (%)	22 (67)	14 (67)	8 (67)	30 (51)	20 (53)	10 (48)
Physiotherapy, n (%)	20 (61)	12 (57)	8 (67)	35 (59)	24 (63)	11 (52)
Social services assessment/support, n (%)	2 (6)	2 (10)	0 (0)	10 (17)	6 (16)	4 (19)
Treatment from CAMHS, n (%)	11 (33)	6 (29)	5 (42)	20 (34)	16 (42)	4 (19)
**Total number of treatments per patient**						
0	4 (12)	3 (14)	1 (8)	6 (10)	3 (8)	3 (25)
1-2	9 (27)	5 (24)	4 (33)	20 (34)	12 (32)	9 (75)
3-4	13 (39)	7 (33)	6 (50)	20 (34)	14 (37)	7 (58)
5-6	7 (21)	5 (24)	2 (17)	9 (15)	9 (24)	2 (17)
7-8	0 (0)	0 (0)	0 (0)	0 (0)	0 (0)	0 (0)
Median (IQR)	3.0 (1.0 - 4.0)			3.0 (2.0 - 4.0)		

Table 3: Management received by CYP with confirmed severe, probable and possible severe ME/CFS, showing those managed in specialist services BACK

Confidential: For Review Only

Contributorship Statement

This study was conceived and designed by EMC and AB. AR, SB performed data collection, analysis and interpretation. AR, KP, SB and II wrote the manuscript. All authors contributed to manuscript revisions, have read the final manuscript and approved it for publication. All authors agree to be accountable for all aspects of the work.

We would like to acknowledge and thank all members of Action for ME and the CFS/ME PAG for their assistance in study design.

Confidential: For Review Only

References

1. NICE. Chronic fatigue syndrome/myalgic encephalomyelitis (or encephalopathy): diagnosis and management of CFS/ME in adults and children. In: Excellence. NIfC, editor. 2021.

2. Carruthers BM, van de Sande MI, De Meirleir KL, Klimas NG, Broderick G, Mitchell T, et al. Myalgic encephalomyelitis: International Consensus Criteria. Journal of Internal Medicine. 2011;270(4):327-38.

3. The Chronic Fatigue Syndrome: A Comprehensive Approach to Its Definition and Study. Annals of Internal Medicine. 1994;121(12):953-9.

4. Sharpe MC, Archard LC, Banatvala JE, Borysiewicz LK, Clare AW, David A, et al. A report--chronic fatigue syndrome: guidelines for research. Journal of the Royal Society of Medicine. 1991;84(2):118-21.

5. Jackson ML, Bruck D. Sleep abnormalities in chronic fatigue syndrome/myalgic encephalomyelitis: a review. Journal of clinical sleep medicine : JCSM : official publication of the American Academy of Sleep Medicine. 2012;8(6):719-28.

6. NICE. Chronic fatigue syndrome/myalgic encephalomyelitis (or encephalopathy): diagnosis and management. NG2062021.

7. Crawley E, Sterne JA. Association between school absence and physical function in paediatric chronic fatigue syndrome/myalgic encephalopathy. Arch Dis Child. 2009;94(10):752-6.

8. Sankey A, Hill CM, Brown J, Quinn L, Fletcher A. A follow-up study of chronic fatigue syndrome in children and adolescents: symptom persistence and school absenteeism. Clin Child Psychol Psychiatry. 2006;11(1):126-38.

9. Kennedy G, Underwood C, Belch JJ. Physical and functional impact of chronic fatigue syndrome/myalgic encephalomyelitis in childhood. Pediatrics. 2010;125(6):e1324–e30.

10. Dowsett EG, Colby J. Long-Term Sickness Absence Due to ME/CFS in UK Schools. Journal of Chronic Fatigue Syndrome. 1997;3(2):29-42.

11. Loades ME, Crawley E, Chalder T, Flannery H. Paediatric chronic fatigue syndrome: 25 year perspective. Clinical Child Psychology and Psychiatry. 2020;26(1):8-17.

12. Royston A, Rai M, Bridgen A, Burge S, Segal T, Crawley E. Severe ME/CFS in children and young people: a British Paediatric Surveillance Unit study. 2022.

13. Crawley E, Hughes R, Northstone K, Tilling K, Emond A, Sterne JA. Chronic disabling fatigue at age 13 and association with family adversity. Pediatrics. 2012;130(1):e71-9.

14. Rimes KA, Goodman R, Hotopf M, Wessely S, Meltzer H, Chalder T. Incidence, Prognosis, and Risk Factors for Fatigue and Chronic Fatigue Syndrome in Adolescents: A Prospective Community Study. Pediatrics. 2007;119(3):e603.

15. Farmer A, Fowler T, Scourfield J, Thapar A. Prevalence of chronic disabling fatigue in children and adolescents. British Journal of Psychiatry. 2004;184(6):477-81.

Confidential: For Review Only

16. Lim EJ, Ahn YC, Jang ES, Lee SW, Lee SH, Son CG. Systematic review and meta-analysis of the prevalence of chronic fatigue syndrome/myalgic encephalomyelitis (CFS/ME). J Transl Med. 2020;18(1):100.

17. Montoya JG, Dowell TG, Mooney AE, Dimmock ME, Chu L. Caring for the Patient with Severe or Very Severe Myalgic Encephalomyelitis/Chronic Fatigue Syndrome. Healthcare. 2021;9(10).

18. Newhart VA, Olson JS, Warschauer M, Eccles JS, editors. Go home and get better: An exploration of inequitable educational services for homebound children2017.

19. McDermott C, Al Haddabi A, Akagi H, Selby M, Cox D, Lewith G. What is the current NHS service provision for patients severely affected by chronic fatigue syndrome/myalgic encephalomyelitis? A national scoping exercise. BMJ Open. 2014;4(6):e005083.

20. Knowles RL, Friend H, Lynn R, Mitchell S, Michie C, Ihekweazu C. Surveillance of rare diseases: a public health evaluation of the British Paediatric Surveillance Unit. Journal of public health (Oxford, England). 2012;34(2):279-86.

21. GOSH. PAEDIATRIC & ADOLESCENT CHRONIC FATIGUE SERVICES - UK 2015 [Available from: https://media.gosh.nhs.uk/documents/Specialist_Paediatric_CFSME_Services_England_updated_May_2015.pdf.

22. BACME. Specialist ME/CFS services map: British Association of Clinicians in ME/CFS; 2018 [Available from: https://bacme.info/services-map/.

23. NICE. Chronic fatigue syndrome/myalgic encephalomyelitis (or encephalopathy): diagnosis and management of chronic fatigue syndrome/myalgic encephalomyelitis (or encephalopathy) in adults and children. In: Turnbull N, Shaw EJ, Baker R, Dunsdon S, Costin N, Britton G, et al., editors. 2007.

24. Pendergrast T, Brown A, Sunnquist M, Jantke R, Newton JL, Strand EB, et al. Housebound versus nonhousebound patients with myalgic encephalomyelitis and chronic fatigue syndrome. Chronic illness. 2016;12(4):292-307.

25. Collard SS, Murphy J. Management of chronic fatigue syndrome/myalgic encephalomyelitis in a pediatric population: A scoping review. Journal of child health care : for professionals working with children in the hospital and community. 2020;24(3):411-31.

26. Gordon BA, Knapman LM, Lubitz L. Graduated exercise training and progressive resistance training in adolescents with chronic fatigue syndrome: a randomized controlled pilot study. Clinical rehabilitation. 2010;24(12):1072-9.

27. Clark LV, Pesola F, Thomas JM, Vergara-Williamson M, Beynon M, White PD. Guided graded exercise self-help plus specialist medical care versus specialist medical care alone for chronic fatigue syndrome (GETSET): a pragmatic randomised controlled trial. The Lancet. 2017;390(10092):363-73.

28. Al-Haggar M, Al-Naggar ZA, Abdel-Salam MA. Biofeedback and cognitive behavioral therapy for Egyptian adolescents suffering from chronic fatigue syndrome. Journal of pediatric neurology. 2015;04:161-9.

Confidential: For Review Only

29. Stulemeijer M, de Jong LW, Fiselier TJ, Hoogveld SW, Bleijenberg G. Cognitive behaviour therapy for adolescents with chronic fatigue syndrome: randomised controlled trial. BMJ (Clinical research ed). 2005;330(7481):14.

30. White PD, Goldsmith KA, Johnson AL, Potts L, Walwyn R, DeCesare JC, et al. Comparison of adaptive pacing therapy, cognitive behaviour therapy, graded exercise therapy, and specialist medical care for chronic fatigue syndrome (PACE): a randomised trial. Lancet. 2011;377(9768):823-36.

31. Brigden A, Beasant L, Gaunt D, Hollingworth W, Mills N, Solomon-Moore E, et al. Results of the feasibility phase of the managed activity graded exercise in teenagers and pre-adolescents (MAGENTA) randomised controlled trial of treatments for chronic fatigue syndrome/myalgic encephalomyelitis. Pilot and Feasibility Studies. 2019;5(1):151.

32. Castro-Marrero J, Sáez-Francàs N, Santillo D, Alegre J. Treatment and management of chronic fatigue syndrome/myalgic encephalomyelitis: all roads lead to Rome. Br J Pharmacol. 2017;174(5):345-69.

33. Kingdon C, Giotas D, Nacul L, Lacerda E. Health Care Responsibility and Compassion-Visiting the Housebound Patient Severely Affected by ME/CFS. Healthcare (Basel, Switzerland). 2020;8(3).

Confidential: For Review Only

BPSU ID: ☐☐ ☐☐☐☐ ☐☐

BPSU Severe CFS/ME Data Collection Form v4.0 31/01/2018
IRAS Project ID: 223838

British Paediatric Surveillance Unit Study

Severe Chronic Fatigue Syndrome/Myalgic Encephalitis

Data Collection Form

CONFIDENTIAL

Confidential: For Re

Section A: Reporter Details

1.1 Date of completion of questionnaire: DD/MM/YYYY

1.2 Consultant responsible for case: ____________________

1.3 Hospital name: ____________________

1.4 Telephone number: ____________ Email: ____________

1.5 Has the patient been referred to/from another centre? Yes ☐ No ☐ Not known ☐

If yes: 1) please name centre: ____________________

2) please name consultant: ____________________

Section B: Case Details

2.1 NHS/CHI No: ☐☐☐ ☐☐☐ ☐☐☐☐

2.2 Hospital No: ☐☐☐☐☐☐☐☐☐☐

2.3 Sex: M ☐ F ☐ Date of birth: DD/MM/YYYY

2.4 Ethnicity*: ☐☐ Specify if any "Other" background

**Please choose the correct ethnicity code from Appendix A overleaf*

2.5 Partial postcode (first three letters) ☐☐☐

BPSU Severe CFS/ME Data Collection Form v4.0 31/01/2018
IRAS Project ID: 223838

BPSU ID: ☐☐ ☐☐☐☐ ☐☐

Appendix A

Appendix A: Coding for Ethnic Group (ONS 2001 for UK / Ireland wide data collection)

		Ethnicity Code	
**A**	**White**	**1**	Any White background
**B**	**Mixed**	**2**	White and Black Caribbean
		**3**	White and Black African
		**4**	White and Asian
		**5**	Any Other Mixed background, *please write in **section***
**C**	**Asian or Asian British**	**6**	Indian
		**7**	Pakistani
		**8**	Bangladeshi
		**9**	Any Other Asian background, *please write in **section***
**D**	**Black or British Black**	**10**	Caribbean
		**11**	African
		**12**	Any Other African background, *please write in **section***
**E**	**Chinese or other ethnic group**	**13**	Chinese
		**14**	Any Other, *please write in **section***
**F**	**Unknown**	**15**	Ethnicity not known

ew Only

BPSU Severe CFS/ME Data Collection Form v4.0 31/01/2018
IRAS Project ID: 223838

Section C: Presentation/Clinical features

Give best possible estimate

3.1 Date when fatigue (as reported by mother/chid or first recorded by medical team) DD/MM/YY

Date of diagnosis DD/MM/YY

3.2 Date when became housebound DD/MM/YY

3.3 Date of most recent medical assessment DD/MM/YY

3.4 **The fatigue:** *(Please tick)*

	Yes	No	Don't know
Is not lifelong (fatigue not noticeable from birth)	☐	☐	☐
Lasted 3 months or longer	☐	☐	☐
Not due to over exertion (for example sport)	☐	☐	☐
Not relieved by rest	☐	☐	☐
Is not because of other medical conditions	☐	☐	☐
Means child unable to leave the house without severe and prolonged after effect.	☐	☐	☐
Means child only leaves house occasionally (less than once a week)	☐	☐	☐
Causes significant reduction in ability to carry out activities of daily living (eating, drinking, preparing food, toileting, washing, walking)	☐	☐	☐

3.5 Does the child have the following symptoms?

	Yes	No	Don't know
Impaired memory or concentration	☐	☐	☐
Post-exertion malaise*	☐	☐	☐
Problems with sleep including un-refreshing sleep	☐	☐	☐
Muscle pain	☐	☐	☐
Pain in multiple joints	☐	☐	☐
Headaches of a new kind or greater severity	☐	☐	☐
Sore throat, frequent or recurring	☐	☐	☐
Tender lymph nodes (cervical or axillary)	☐	☐	☐

* physical or mental exertions bring on "extreme, prolonged exhaustion and sickness"

Section D: Function

Give best possible estimate

4.1 What has the child's school attendance been over the last 6 weeks? (Please tick best estimate)

	Yes
No tuition (no home tuition or schooling)	☐
Home tuition	☐
Attending school or hospital school for less than one hour a week	☐
Attending school for more than one hour a week	☐
Don't know	☐

***if it has been the school holidays please use equivalent activity**

4.2 What is the child's current level of independence in activities of daily living?" (eating, drinking, preparing food, toileting, washing, walking)

	Yes
Requires help with all activities of daily living	☐
Requires some help with daily activities sometimes	☐
Able to do all daily activities over last 6 weeks	☐

Please provide further information:

What help/support is required?

BPSU Severe CFS/ME Data Collection Form v4.0 31/01/2018
IRAS Project ID: 223838

Section D: Investigations and Management

5.1 Are the following test results in the normal range based on your local lab ranges?

	Yes	No	Not done or don't know
full blood count,	☐	☐	☐
urea and electrolytes	☐	☐	☐
serum creatinine	☐	☐	☐
thyroid function	☐	☐	☐
erythrocyte sedimentation rate or plasma viscosity	☐	☐	☐
C-reactive protein	☐	☐	☐
random blood glucose	☐	☐	☐
screening blood tests for gluten sensitivity	☐	☐	☐
serum calcium	☐	☐	☐
creatine kinase	☐	☐	☐
serum ferritin	☐	☐	☐
liver function	☐	☐	☐
Urinalysis	☐	☐	☐

5.2 Has the child received any of the following? (please tick all that apply)

	Yes	No	Don't Know
Cognitive Behaviour Therapy	☐	☐	☐
Graded Exercise Therapy	☐	☐	☐
Activity Management	☐	☐	☐
Medication for symptoms	☐	☐	☐
Domiciliary assessment or treatment	☐	☐	☐
Treatment from CAMHS?	☐	☐	☐
Physiotherapy?	☐	☐	☐
Social services assessment/support?	☐	☐	☐

BPSU Severe CFS/ME Data Collection Form v4.0 31/01/2018
IRAS Project ID: 223838

Thank you for taking the time to complete the Questionnaire

Please print and return the completed form in the SAE to:

Professor Esther Crawley

1-5 Whiteladies Road

Clifton, Bristol, BS8 1NU

If you have any questions about the study please do not hesitate to contact the investigators by email or telephone:

Telephone: ____________________ Email: ______________________________

Confidential: For Review Only

BPSU Severe CFS/ME Data Collection Form v4.0 31/01/2018
IRAS Project ID: 223838

BPSU ID: ☐☐ ☐☐☐☐ ☐☐

British Paediatric Surveillance Unit Study Severe Chronic Fatigue Syndrome/Myalgic Encephalitis

Data Collection Form

CONFIDENTIAL

Confidential: For Revi

Section A: Reporter Details

1.1 Date of completion of questionnaire: DD/MM/YYYY

1.2 Consultant responsible for case: ______

1.3 Hospital name: ______

1.4 Telephone number: ______ Email: ______

1.5 Has the patient been referred to/from another centre? Yes ☐ No ☐ Not known ☐

If yes: 1) please name centre: ______

2) please name consultant: ______

Section B: Case Details

2.1 NHS/CHI No: ☐☐☐ ☐☐☐ ☐☐☐☐

2.2 Hospital No: ☐☐☐☐☐☐☐☐☐☐

2.3 Sex: M ☐ F ☐ Date of birth: DD/MM/YYYY

2.4 Ethnicity*: ☐☐ Specify if any "Other" background

**Please choose the correct ethnicity code from Appendix A overleaf*

2.5 Partial postcode (first three letters) ☐☐☐

BPSU Severe CFS/ME Data Collection Form v4.0 31/01/2018
IRAS Project ID: 223838

BPSU ID:

Appendix A

Appendix A: Coding for Ethnic Group (ONS 2001 for UK / Ireland wide data collection)

! !	"#$%&'&#(!)*+,!	
-! .$&#,	/!	Any White background
0! 1&2,+	3!	White and Black Caribbean
!	4!	White and Black African
!	5!	White and Asian
!	6!	Any Other Mixed background, *please write in* ***section***
)! -7&8%!*9!-7&8%!09&#&7$	:!	Indian
!	;!	Pakistani
!	<!	Bangladeshi
!	=!	Any Other Asian background, *please write in* ***section***
>! 0?8'@!*9!09&#&7$!0?8'@	/A!	Caribbean
!	//!	African
!	/3!	Any Other African background, *please write in* ***section***
"!)$&%,7,!*9!*#$,9! ,#$%&'!B9*CD	/4!	Chinese
!	/5!	Any Other, *please write in* ***section***
E! F%@%*G%!	/6!	Ethnicity not known

ew Only

BPSU Severe CFS/ME Data Collection Form v4.0 31/01/2018
IRAS Project ID: 223838

Section C: Presentation/Clinical features

Give best possible estimate

3.1 Date when fatigue (as reported by mother/chid or first recorded by medical team) DD/MM/YY

Date of diagnosis DD/MM/YY

3.2 Date when became housebound DD/MM/YY

3.3 Date of most recent medical assessment DD/MM/YY

3.4 **The fatigue:** *(Please tick)*

	Yes	No	Don't know
Is not lifelong (fatigue not noticeable from birth)	☐	☐	☐
Lasted 3 months or longer	☐	☐	☐
Not due to over exertion (for example sport)	☐	☐	☐
Not relieved by rest	☐	☐	☐
Is not because of other medical conditions	☐	☐	☐
Means child unable to leave the house without severe and prolonged after effect.	☐	☐	☐
Means child only leaves house occasionally (less than once a week)	☐	☐	☐
Causes significant reduction in ability to carry out activities of daily living (eating, drinking, preparing food, toileting, washing, walking)	☐	☐	☐

!

3.5 Does the child have the following symptoms?

	Yes	No	Don't know
Impaired memory or concentration	☐	☐	☐
Post-exertion malaise*	☐	☐	☐
Problems with sleep including un-refreshing sleep	☐	☐	☐
Muscle pain	☐	☐	☐
Pain in multiple joints	☐	☐	☐
Headaches of a new kind or greater severity	☐	☐	☐
Sore throat, frequent or recurring	☐	☐	☐
Tender lymph nodes (cervical or axillary)	☐	☐	☐

* physical or mental exertions bring on "extreme, prolonged exhaustion and sickness"

BPSU Severe CFS/ME Data Collection Form v4.0 31/01/2018
IRAS Project ID: 223838

Section D: Function

Give best possible estimate

4.1 What has the child's school attendance been over the last 6 weeks? (Please tick best estimate)

	Yes
No tuition (no home tuition or schooling)	☐
Home tuition	☐
Attending school or hospital school for less than one hour a week	☐
Attending school for more than one hour a week	☐
Don't know	☐

H&I!&#!$87!J,,%!#$,!7'$**?!$*?&+8(7!D?,87,!C7,!,KC&L8?,%#!8'#&L&#(!

4.2 What is the child's current level of independence in activities of daily living?" (eating, drinking, preparing food, toileting, washing, walking)

	Yes
Requires help with all activities of daily living	☐
Requires some help with daily activities sometimes	☐
Able to do all daily activities over last 6 weeks	☐

Please provide further information:

BPSU Severe CFS/ME Data Collection Form v4.0 31/01/2018
IRAS Project ID: 223838

Section D: Investigations and Management

5.1 Are the following test results in the normal range based on your local lab ranges?

	Yes	No	Not done or don't know
full blood count,	☐	☐	☐
urea and electrolytes	☐	☐	☐
serum creatinine	☐	☐	☐
thyroid function	☐	☐	☐
erythrocyte sedimentation rate or plasma viscosity	☐	☐	☐
C-reactive protein	☐	☐	☐
random blood glucose	☐	☐	☐
screening blood tests for gluten sensitivity	☐	☐	☐
serum calcium	☐	☐	☐
creatine kinase	☐	☐	☐
serum ferritin	☐	☐	☐
liver function	☐	☐	☐
Urinalysis	☐	☐	☐

5.2 Has the child received any of the following? (please tick all that apply)

	Yes	No	Don't Know
Cognitive Behaviour Therapy	☐	☐	☐
Graded Exercise Therapy	☐	☐	☐
Activity Management	☐	☐	☐
Medication for symptoms	☐	☐	☐
Domiciliary assessment or treatment	☐	☐	☐
Treatment from CAMHS?	☐	☐	☐
Physiotherapy?	☐	☐	☐
Social services assessment/support?	☐	☐	☐

BPSU Severe CFS/ME Data Collection Form v4.0 31/01/2018
IRAS Project ID: 223838

Thank you for taking the time to complete the Questionnaire

Please print and return the completed form in the SAE to:

!

!

!

If you have any questions about the study please do not hesitate to contact the investigators by email or telephone:

Telephone: ! Email:

Confidential: For Review Only

BMJ Paediatrics Open

Management of Severe ME/CFS in Children and Young People in the UK: a British Paediatric Surveillance Unit study

Journal:	*BMJ Paediatrics Open*
Manuscript ID	bmjpo-2023-002436.R1
Article Type:	Original research
Date Submitted by the Author:	21-Feb-2024

Complete List of Authors:	Royston, Alexander; University of Bristol Medical School, Centre for Academic Child Health; University Hospitals Bristol and Weston NHS Foundation Trust<br>Burge, Sarah; University of Bristol Medical School, Centre for Academic Child Health<br>Idini, Ilaria; Royal United Hospitals Bath NHS Foundation Trust<br>Brigden, Amberly; University of Bristol, School of Engineering Mathematics and Technology<br>Pike, Katharine; University of Bristol Medical School, Centre for Academic Child Health

Keywords:	Chronic Fatigue Syndrome, Epidemiology, Adolescent Health

SCHOLARONE™
Manuscripts

I, the Submitting Author has the right to grant and does grant on behalf of all authors of the Work (as defined in the below author licence), an exclusive licence and/or a non-exclusive licence for contributions from authors who are: i) UK Crown employees; ii) where BMJ has agreed a CC-BY licence shall apply, and/or iii) in accordance with the terms applicable for US Federal Government officers or employees acting as part of their official duties; on a worldwide, perpetual, irrevocable, royalty-free basis to BMJ Publishing Group Ltd ("BMJ") its licensees and where the relevant Journal is co-owned by BMJ to the co-owners of the Journal, to publish the Work in this journal and any other BMJ products and to exploit all rights, as set out in our licence.

The Submitting Author accepts and understands that any supply made under these terms is made by BMJ to the Submitting Author unless you are acting as an employee on behalf of your employer or a postgraduate student of an affiliated institution which is paying any applicable article publishing charge ("APC") for Open Access articles. Where the Submitting Author wishes to make the Work available on an Open Access basis (and intends to pay the relevant APC), the terms of reuse of such Open Access shall be governed by a Creative Commons licence – details of these licences and which Creative Commons licence will apply to this Work are set out in our licence referred to above.

Other than as permitted in any relevant BMJ Author's Self Archiving Policies, I confirm this Work has not been accepted for publication elsewhere, is not being considered for publication elsewhere and does not duplicate material already published. I confirm all authors consent to publication of this Work and authorise the granting of this licence.

Confidential: For Review Only

Management of Severe ME/CFS in Children and Young People in the UK: a British Paediatric Surveillance Unit study

Authors:

Royston[1], Alexander Peter

Burge[1], Sarah

Idini[2], Ilaria

Brigden[3], Amberly

Pike[1], Katharine Claire

Journal

1) BMJ Paediatrics Open

Author affiliations

[1] Centre for Academic Child Health, Bristol Medical School, Canynge Hall, 39 Whatley Road, Bristol BS8 2PS, UK

[2] Royal United Hospitals Bath NHS Foundation Trust, Combe Park, Bath BA1 3NG, UK

[3] School of Engineering Mathematics and Technology, University of Bristol, Bristol, BS8 1UB, UK

Corresponding author:

Dr Alexander Royston

ar16623@bristol.ac.uk

Key words

Paediatric, ME/CFS, chronic fatigue syndrome

Confidential: For Review Only

ABSTRACT

Objective: Severe myalgic encephalomyelitis or chronic fatigue syndrome (ME/CFS) in children and young people (CYP) is a little understood condition which significantly impacts education, development, and quality of life. We used data from a population-wide surveillance study to explore the screening investigation, referral, and management of suspected cases of paediatric severe ME/CFS.

Methods: A British Paediatric Surveillance Unit (BPSU) study reported cases of CYP with suspected severe ME/CFS between February 2018 and February 2019. Paediatricians reporting cases to BPSU and allied healthcare professionals in two large specialist paediatric ME/CFS centres were invited to complete questionnaires for CYP meeting the surveillance case definition. The study focussed primarily on CYP with confirmed severe ME/CFS and the extent to which their care met NICE recommendations, but also considered separately those with probable or possible severe ME/CFS.

Results: This study incudes a total of 92 CYP with suspected severe ME/CFS; 33 meeting criteria for severe ME/CFS and an additional 59 classified as probable or possible severe ME/CFS. For 16 possible cases incomplete investigation to exclude alternative diagnoses prevented confirmation of a severe ME/CFS diagnosis. Only 21 of 33 (64%) confirmed severe ME/CFS cases had been referred to specialist services. The management provided varied considerably between patients and four received nothing at all. Of the management provided, the most frequent approaches were medication (67%), activity management (61%) and physiotherapy (61%). Domiciliary assessments and support, and social services referrals were received by 12% and 6% of confirmed severe cases. Similar proportions of management approaches were seen in probable/possible severe ME/CFS.

Conclusions: Full investigation is frequently incomplete in CYP with suspected severe ME/CFS and recommendations for referral and management are poorly implemented, in particular the needs of CYP who are unable to leave their home might be poorly met.

Confidential: For Review Only

Key Messages

What is already known on this topic:

- Myalgic Encephalomyelitis, also known as Chronic Fatigue Syndrome (ME/CFS), is a disabling condition that affects people of all ages, including children and young people (CYP).
- NICE published guidelines in 2021 recommending that CYP with suspected severe ME/CFS should have standardised screening investigations to exclude conditions other than ME/CFS that might cause similar symptoms.
- Those with a confirmed diagnosis should be referred to specialist care and this should include domiciliary visits, particularly for individuals unable to leave their homes. It is not known how often these recommendations are implemented.

What this study adds

- The standardised investigations recommended by NICE are inconsistently performed for CYP with suspected severe ME/CFS, and this is a barrier to confirmation of diagnosis.
- Referral guidelines are not met for nearly one third of CYP diagnosed with severe ME/CFS.
- Medication to manage symptoms are prescribed for the majority but domiciliary visits and social service referrals are quite uncommon, received by only 12% of confirmed cases.

How this study might affect research, practice or policy

- This study suggests the provision for CYP with ME/CFS, including appropriate investigation and onward referral to specialist services requires standardisation in clinical practise.
- Further research is required to identify barriers to the NICE guideline's implementation and how these can be tackled.

Confidential: For Review Only

SUMMARY BOX

A funding statement:

The study was funded by The National Institute for Health Research (Senior Research Fellowship, SRF-2013-06-013) and a grant from the Royal United Hospitals Bath NHS Foundation Trust.

Disclosure statement: The authors have declared no conflicts of interests.

Any checklist/flow diagram for the appropriate reporting statement: STROBE

Patient consent form: not included – please see CAG Section 251 approval statement in Methods.

Please provide a data sharing statement: Data unavailable for review due to being covered by CAG Section 251 approval **(18/CAG/0052)**

Confidential: For Review Only

INTRODUCTION

Myalgic Encephalomyelitis/Chronic Fatigue Syndrome (ME/CFS) is a disabling illness of unclear aetiology. There are several diagnostic frameworks for ME/CFS(1-4); each describes symptoms including fatigue, post exertional malaise (PEM)(5), muscle and joint pain, disturbed sleeping(5), and difficulties concentrating(6). Severely affected individuals can have limited mobility, be unable to leave their home and have severe cognitive difficulties. ME/CFS can have a major impact on children and young people's (CYP) development and functioning. Reduced school attendance is a particularly significant problem(7), impacting on development, education and career plans(8-10).

As recently as the 1990s, the prevalence of ME/CFS in teenagers was unknown and there was little acknowledgement that primary school-age children can be affected(11). A recent surveillance study conducted in the United Kingdom (UK) and Republic of Ireland (RoI) reported 33 confirmed severe ME/CFS cases in CYP between 5 and 16 years of age, suggesting a prevalence of 3.18 per million children (95%CI 2.19 to 4.47)(12). However, epidemiological prevalence estimates vary depending upon the diagnostic criteria and methodology used and upon whether thorough clinical investigation has been completed to confirm a diagnosis. The 2021 NICE guidelines include recommendations about screening investigations necessary to exclude alternative causes of fatigue(13-16).

NICE recommends referral to a specialist centre in order to confirm diagnosis and develop a care and support plan. Since CYP with severe ME/CFS can be unable to leave their home, it can be difficult for them to attend outpatient clinics and in the absence of outreach services their health needs may not be met(17, 18). NICE guidelines recommend home visits to conduct holistic assessments to enable the development of care and support plans by a ME/CFS specialist team(6).

A scoping study conducted of UK services treating adults with severe ME/CFS reported that 55% (27 of 49) met NICE guidelines for treatment of severe ME/CFS and 12% (6 of 49) offered

Confidential: For Review Only

occasional support where funding allowed. However, 33% (16 of 49) of ME/CFS services provided no service for housebound patients(19). No equivalent study has been conducted in CYP and it is currently difficult to evaluate or plan service provision for this population.

We aimed to explore whether the condition is fully investigated in the UK and Republic Ireland (as per NICE guidelines), whether appropriate referrals are made to specialist services and the range of managements delivered.

Confidential: For Review Only

METHOD

Study Design

Data was collected using a prospective cross-sectional questionnaire. Using the BPSU's(20) reporting system for rare conditions(12), between February 2018 and February 2019 paediatricians in the UK and Republic of Ireland were invited to report 5 - 16-year-old CYP, meeting a clinical diagnosis of ME/CFS who due to fatigue were able to attend school for an hour or less per week over the last six term-time weeks. Paediatricians reporting cases were asked to complete detailed questionnaires on the cases that they reported. Along with patient demographics, symptomology and function, the questionnaire asked whether the NICE-recommended screening investigations were completed, whether the CYP had been referred to a specialist centre, and about the management they received. This study also employed parallel reporting from two large specialist paediatric ME/CFS centres (the Royal United Hospitals Bath NHS Foundation Trust and University College London Hospital, London). In these centres CYP are assessed and treated by allied healthcare professionals who do not receive BPSU survey requests. Questionnaires were distributed to professionals working in the RUH and UCLH ME/CFS centres to complete for CYP under management at that centre meeting the surveillance case definition.

Case Definition

A staged process was used to confirm severe ME/CFS; this required screening blood tests to exclude alternative diagnoses, and assessment of symptom characteristics, duration and impact(12). Patients with abnormal blood tests suggestive of another cause for the fatigue were classified as not severe ME/CFS and excluded from further analysis. Urinalysis is recommended within the NICE guideline, but this was removed as a mandatory criterion by the study team because it was so often missed. Patients otherwise meeting the case definition but for whom it was unclear whether they had experienced a significant reduction in activities of daily living were classified as having probable severe ME/CFS. Those meeting most criteria

Confidential: For Review Only

but with incomplete screening tests or information on function were classified as having possible severe ME/CFS.

In the absence of an official paediatric ME/CFS specialist service specification, a list of specialist services was compiled from services containing a paediatrician and treating CYP that were named either in a list published by Great Ormond Street Hospital for Children(21) or the 2018 directory(22) of the British Association of Clinicians in ME/CFS (BACME). The questionnaire asked whether the CYP had received any of the following treatments/assessments: activity management, cognitive behaviour therapy (CBT), domiciliary assessment or treatment, graded exercise therapy, medication for symptoms, physiotherapy, social services assessment and support, or treatment from Child and Adolescent mental Health Services (CAMHS). These approaches were in line with NICE guidelines at the time of study design(23), though more recent guidelines also recommend dietetic assessments for certain CYP and prohibit graded exercise therapy(6).

Data Analysis

Data were analysed using Stata V16.0 (Statcorp, Tx) and expressed using appropriate summary statistics. The primary analysis focused upon identifying the proportion of CYP with confirmed severe ME/CFS that were appropriately referred to specialist services and reporting the treatments offered to this group. Secondary analyses reported 1) demographics, referral, and management of children with probable or possible severe ME/CFS and 2) screening investigations completed in CYP with possible severe ME/CFS.

Patient and Public Involvement

The study idea and its proposal were developed with Action for ME and the CFS/ME Patient Advisory Group was both involved in the study design and writing public information leaflets.

Ethical approval

Confidential: For Review Only

This study was approved by the BPSU Scientific Committee; National Research Ethics System Committee South-West-Central Bristol Research Ethics Committee (Ref: 18/SW/0051; IRAS ID: 223838); Section 251 Confidentiality Advisory Group permission (Ref: 18/CAG/00051); and Public Benefit and Privacy Panel for Health and Social Care approval (Ref: 1718-0341).

Confidential: For Review Only

Confidential: For Review Only

RESULTS

Questionnaires were completed for 92 CYP with a clinical diagnosis of ME/CFS significantly impacting school attendance. Sixty-four cases were identified from the BPSU surveillance survey and 28 from the RUH and UCLH specialist centres. Severe ME/CFS was confirmed for 33 CYP. The functional criterion was not met, or functional status or investigation data were missing in 59 cases, which were defined as probable or possible severe ME/CFS. Nineteen of 33 confirmed severe cases (58%) were female, 32 (97%) were white and median age of fatigue onset was 13.0 years [IQR 11.4, 14.5](Table 1). Demographic characteristics were similar in the possible/probable cases(Table 1).

Screening investigations

To meet the case definition of confirmed severe ME/CFS all investigations were complete and reported as normal in confirmed severe ME/CFS cases, (except for urinalysis in 11 cases, (33%))(Table 1). Of the possible/probable cases, only 15 cases had complete investigations. The proportion reporting a blood result varied by test. Urea, electrolytes and creatinine, and full blood count were the most commonly reported (86%), whilst random blood glucose and creatine kinase were less reported (64% and 67%, respectively). Urinalysis was completed and reported as normal in 14 CYP (26%).

Referral

Only 64% (21/33) of patients with confirmed severe ME/CFS had been referred to a specialist paediatric centre, 11 (33%) after referral from a non-specialist paediatrician and 10 (30%) after referral from Primary Care(Table 2). Of the 12 CYP with confirmed severe ME/CFS not referred to a specialist centre, three were referred between centres classified in this study as non-specialist.

In possible/probable cases, 30 (51%) were referred from a paediatrician to a specialist centre and 8 (14%) were seen by Specialist centres directly following referral from Primary Care. This left 21 possible/probable cases (36%) reported by non-specialists and not referred to a specialist centre, 3 (6%) of whom were referred between non-specialist centres(Table 2).

Management

Amongst CYP with confirmed severe ME/CFS, 27 had received between one and six management approaches (median 3, IQR 1-4) but 4/33 received no management of any kind(Table 3). The commonest intervention was medication (22/33: 67%). Few specific examples of medication were given, though melatonin, laxatives and thyroxine were described in free-text responses. Activity management and physiotherapy were the second most used approaches (both used in 61%), whilst fewer CYP received treatment from CAMHS or CBT (33% and 24%, respectively). Only 4/33 (12%) of severe cases had received a domiciliary visit and 2/33 (6%) social service assessment or support.

Forty-nine (83%) CYP with possible/probable severe ME/CFS received between one and six management approaches (median 3, IQR 2, 4), whilst six (10%) received no management(Table 3). The proportion receiving physiotherapy was similar in the possible/probable group to that in those with a confirmed diagnosis (59%), though a slightly lower proportion received medication (30/59, 51%) and higher proportion received activity management (69%). The proportion of children in the probable/possible group who received CAMHS and CBT was similar to that seen in CYP with a confirmed diagnosis. Small numbers received either domiciliary assessment or treatment or social services assessment or support (6/59 (10%) and 10/59 (17%), respectively).

Confidential: For Review Only

A lower proportion of CYP with confirmed or possible/probable severe ME/CFS seen in specialist centres received activity management or physiotherapy compared with CYP seen in non-specialist centres. The proportion of patients offered specific management approaches appeared similar for those with confirmed or possible/probable severe ME/CFS(Table 3). The proportion receiving domiciliary assessment/treatment or social services support was not similar between patients referred or not referred to specialist centres (14% vs 8% [confirmed] and 16% vs 0% [possible/probable]), although numbers are too small to draw definite conclusions from this.

Confidential: For Review Only

DISCUSSION

This study has found that the majority of 92 CYP with suspected severe ME/CFS identified via a UK and RoI surveillance study and survey of two large specialist centres did not receive the screening investigations recommended within current NICE guidelines. Severe ME/CFS was confirmed in 33 (36%) cases but up to a further 16 cases might have been confirmed had blood investigations been completed and reported normal. A substantial number of confirmed severe cases of ME/CFS, 12/33 (36%), and similar proportions of probable or possible cases, were not referred to a specialist centre. The median number of management approaches received was three; and whilst medication (67%), activity management (61%) and physiotherapy (61%) were commonly reported, fewer patients received treatment from CAMHS 11/33 (33%) or CBT 8/33 (24%), and domiciliary assessments; and social services support were received by only 12% and 6% of confirmed severe ME/CFS cases.

Strengths and limitations

The principal strength of this study is that it is a prospective surveillance study using the BPSU's well-established methodology, which generally achieves a good coverage of paediatricians with response rates in the range of 90%. However, potential limitations arose from the challenges of completing a surveillance study during the COVID-19 pandemic, difficulties were encountered in contacting clinicians and in encouraging questionnaire completion and return. Whilst cases identified by the surveillance study overlapped with those identified from the RUH and UCLH centres, the number of duplicates was low which might suggest case identification was incomplete. Our method potentially underestimates prevalence of confirmed severe ME/CFS compared to other population-based studies due to the strict case definition criteria used. However, investigation, referral and management characteristics were reported relating to possible and probable cases which might be included under alternative case definitions. Since a high proportion of patients with severe ME/CFS are housebound, making them 'hard to reach' and potentially underdiagnosed with poor access to support, this might limit the generalisability of this study's findings across the CYP severe ME/CFS population(24).

Comparison with previous studies

Confidential: For Review Only

To our knowledge, this is the first national study carried out in children. A national scoping exercise of UK adults(19), found that whilst 55% of specialist adult ME/CFS centres met NICE guidelines for service provision for patients with severe ME/CFS, but 33% (16/49) provided no service for housebound patients. The most offered management modalities were activity management, CBT and graded activity. The adult study focussed on service provision at the level of specialist centres rather than services provided to individual patients meeting severe ME/CFS criteria.

Although NICE has made recommendations, the evidence for specific management approaches in CYP with severe ME/CFS is scant. Whilst many studies have explored management of ME/CFS in paediatric populations(25), for example evaluations of exercise therapy(26, 27) and CBT(28, 29), none have focused on the severe form of the condition. Inclusion criteria typically either broaden the study samples to include those with milder disease (for example choosing a lower cut-off SF-36 score of 60 for inclusion(30)) or exclude those with severe fatigue ((31)). Generally, trials of approaches that have shown some promise in treating ME/CFS report only limited evidence of efficacy in the most severely ill adult patients(32) and research is lacking in patients of all ages.

Implications

The finding that a substantial number of CYP with suspected severe ME/CFS have incomplete screening investigations not only affects the accuracy of prevalence estimates but also has diagnostic implications. Treatable conditions that resemble ME/CFS might not be identified, and this can lead to inappropriate management. This potential for harm is recognised in the NICE guidance and underlies the recommendation that prescribing should be initiated under the supervision of a paediatrician with expertise in ME/CFS(6). This is a particular concern given that a sizeable proportion of CYP are either without a confirmed diagnosis or under management in non-specialist centres. NICE's 2021 guidance for ME/CFS of all grades of severity, including those with severe forms of the condition(6), recommends clinicians "refer children and young people who have been diagnosed with ME/CFS after assessment by a paediatrician directly to a paediatric ME/CFS specialist team".

Confidential: For Review Only

Our results show that only two thirds of patients with severe ME/CFS were under specialist management when receiving physiotherapy and activity management. NICE recommends specialist physiotherapy advice and additional care for those with severe or very severe ME/CFS, when an approach involving energy management has been chosen(6). Whilst it is encouraging that CYP with severe ME/CFS have access to physiotherapy, it is possible that they may not be receiving optimal care. It is not known why specialist centres appear to recommend activity management and physiotherapy less often.

The scarcity of domiciliary visits is of concern for this patient-group(33). The proportion of patients receiving home assessments (12%) falls short of NICE's recommendations that home visits should be offered to all with severe or very severe ME/CFS(6). Social services assessment and support was similarly rare in our cohort (6%), raising the possibility that CYP and their parents or carers are not receiving the support that they are entitled to.

Confidential: For Review Only

CONCLUSION

Investigation, referral, and management of CYP with suspected severe ME/CFS do not always meet NICE recommendations. This suggests patients may be receiving suboptimal care since alternative diagnoses are not appropriately excluded and specialist care not provided. The low number of CYP receiving domiciliary visits or social services assessment/support limits their access to management. Further work should focus, not only on determining the most effective treatments for children with severe ME/CFS, but also on exploring the barriers to accessing these services and how these can be addressed.

Confidential: For Review Only

Characteristics	Severe ME/CFS		Possible/Probable ME/CFS		
	n=33			*n*	
Female, n (%)	19	(58)	31	58	(54)
Ethnicity A1, n (%)	32	(97)	54	56	(96)
Age at diagnosis, median (IQR)	13.0	(11.4, 14.5)	14.3	50	(13.1, 15.4)
**Reporting method**					
BPSU, n (%)	21	(64)	43	59	(73)
Directly reported from Specialist centre	12	(36)	16	59	(27)
**Investigations**					
Patients with all diagnostic investigations complete, n (%)	33	(100)	35	59	(59)
Patients with all investigations required for management complete, n (%)	33	(100)	41	59	(70)

Table 1: Demographics and investigations for CYP with severe and possible/probable ME/CFS BACK

Reporting and Referrals	Severe ME/CFS		Possible/Probable ME/CFS	
	n=33		*n=59*	
**Referrals**				
From paediatrician to Specialist centre, n (%)	11	(33)	30	(51)
Seen by Specialist centre directly, n (%)	10	(30)	8	(14)
**Total:** seen by Specialist services	21	(64)	38	(64)
Referred between paediatric services (non-specialist), n (%)	3	(9)	3	(5)
Not referred	9	(27)	18	(31)
**Total:** not seen in Specialist services	12	(36)	21	(36)

Table 2: Reporting and referrals of CYP with severe and possible/probable ME/CFS BACK

Management/Treatments	Severe ME/CFS			Possible/Probable ME/CFS		
	Total n=33 (%)	*Spec n=21 (%)*	*Non-spec n=12 (%)*	*Total* n=59 (%)	*Spec n=38 (%)*	*Non-spec n=12 (%)*
Activity Management, n (%)	20 (61)	13 (62)	7 (58)	41 (69)	25 (66)	16 (76)
Cognitive Behavioural Therapy, n (%)	8 (24)	6 (29)	2 (17)	10 (17)	9 (24)	1 (5)
Domiciliary assessment/treatment, n (%)	4 (12)	3 (14)	1 (8)	6 (10)	6 (16)	0 (0)
Graded Exercise Therapy, n (%)	11 (33)	7 (33)	4 (33)	18 (31)	13 (34)	5 (24)
Medication for Symptoms, n (%)	22 (67)	14 (67)	8 (67)	30 (51)	20 (53)	10 (48)
Physiotherapy, n (%)	20 (61)	12 (57)	8 (67)	35 (59)	24 (63)	11 (52)
Social services assessment/support, n (%)	2 (6)	2 (10)	0 (0)	10 (17)	6 (16)	4 (19)
Treatment from CAMHS, n (%)	11 (33)	6 (29)	5 (42)	20 (34)	16 (42)	4 (19)
**Total number of treatments per patient**						
0	4 (12)	3 (14)	1 (8)	6 (10)	3 (8)	3 (25)
1-2	9 (27)	5 (24)	4 (33)	20 (34)	12 (32)	9 (75)
3-4	13 (39)	7 (33)	6 (50)	20 (34)	14 (37)	7 (58)
5-6	7 (21)	5 (24)	2 (17)	9 (15)	9 (24)	2 (17)
7-8	0 (0)	0 (0)	0 (0)	0 (0)	0 (0)	0 (0)
Median (IQR)	3.0 (1.0 - 4.0)			3.0 (2.0 - 4.0)		

Table 3: Management received by CYP with confirmed severe, probable, and possible severe ME/CFS, showing those managed in specialist services BACK

Confidential: For Review Only

Contributorship Statement

This study was conceived and designed by EMC and AB. AR, SB performed data collection, analysis and interpretation. AR, KP, SB and II wrote the manuscript. All authors contributed to manuscript revisions, have read the final manuscript, and approved it for publication. All authors agree to be accountable for all aspects of the work.

We would like to acknowledge and thank all members of Action for ME and the CFS/ME PAG for their assistance in study design.

Confidential: For Review Only

References

1. Carruthers BM, van de Sande MI, De Meirleir KL, Klimas NG, Broderick G, Mitchell T, et al. Myalgic encephalomyelitis: International Consensus Criteria. Journal of Internal Medicine. 2011;270(4):327-38.

2. The Chronic Fatigue Syndrome: A Comprehensive Approach to Its Definition and Study. Annals of Internal Medicine. 1994;121(12):953-9.

3. NICE. Chronic fatigue syndrome/myalgic encephalomyelitis (or encephalopathy): diagnosis and management of CFS/ME in adults and children. In: Excellence. NIfC, editor. 2021.

4. Sharpe MC, Archard LC, Banatvala JE, Borysiewicz LK, Clare AW, David A, et al. A report--chronic fatigue syndrome: guidelines for research. Journal of the Royal Society of Medicine. 1991;84(2):118-21.

5. Jackson ML, Bruck D. Sleep abnormalities in chronic fatigue syndrome/myalgic encephalomyelitis: a review. Journal of clinical sleep medicine : JCSM : official publication of the American Academy of Sleep Medicine. 2012;8(6):719-28.

6. NICE. Chronic fatigue syndrome/myalgic encephalomyelitis (or encephalopathy): diagnosis and management. NG2062021.

7. Crawley E, Sterne JA. Association between school absence and physical function in paediatric chronic fatigue syndrome/myalgic encephalopathy. Arch Dis Child. 2009;94(10):752-6.

8. Sankey A, Hill CM, Brown J, Quinn L, Fletcher A. A follow-up study of chronic fatigue syndrome in children and adolescents: symptom persistence and school absenteeism. Clin Child Psychol Psychiatry. 2006;11(1):126-38.

9. Kennedy G, Underwood C, Belch JJ. Physical and functional impact of chronic fatigue syndrome/myalgic encephalomyelitis in childhood. Pediatrics. 2010;125(6):e1324–e30.

10. Dowsett EG, Colby J. Long-Term Sickness Absence Due to ME/CFS in UK Schools. Journal of Chronic Fatigue Syndrome. 1997;3(2):29-42.

11. Loades ME, Crawley E, Chalder T, Flannery H. Paediatric chronic fatigue syndrome: 25 year perspective. Clinical Child Psychology and Psychiatry. 2020;26(1):8-17.

12. Royston A, Rai M, Bridgen A, Burge S, Segal T, Crawley E. Severe ME/CFS in children and young people: a British Paediatric Surveillance Unit study. 2022.

13. Crawley E, Hughes R, Northstone K, Tilling K, Emond A, Sterne JA. Chronic disabling fatigue at age 13 and association with family adversity. Pediatrics. 2012;130(1):e71-9.

14. Rimes KA, Goodman R, Hotopf M, Wessely S, Meltzer H, Chalder T. Incidence, Prognosis, and Risk Factors for Fatigue and Chronic Fatigue Syndrome in Adolescents: A Prospective Community Study. Pediatrics. 2007;119(3):e603.

15. Farmer A, Fowler T, Scourfield J, Thapar A. Prevalence of chronic disabling fatigue in children and adolescents. British Journal of Psychiatry. 2004;184(6):477-81.

Confidential: For Review Only

16. Lim EJ, Ahn YC, Jang ES, Lee SW, Lee SH, Son CG. Systematic review and meta-analysis of the prevalence of chronic fatigue syndrome/myalgic encephalomyelitis (CFS/ME). J Transl Med. 2020;18(1):100.

17. Montoya JG, Dowell TG, Mooney AE, Dimmock ME, Chu L. Caring for the Patient with Severe or Very Severe Myalgic Encephalomyelitis/Chronic Fatigue Syndrome. Healthcare. 2021;9(10).

18. Newhart VA, Olson JS, Warschauer M, Eccles JS, editors. Go home and get better: An exploration of inequitable educational services for homebound children2017.

19. McDermott C, Al Haddabi A, Akagi H, Selby M, Cox D, Lewith G. What is the current NHS service provision for patients severely affected by chronic fatigue syndrome/myalgic encephalomyelitis? A national scoping exercise. BMJ Open. 2014;4(6):e005083.

20. Knowles RL, Friend H, Lynn R, Mitchell S, Michie C, Ihekweazu C. Surveillance of rare diseases: a public health evaluation of the British Paediatric Surveillance Unit. Journal of public health (Oxford, England). 2012;34(2):279-86.

21. GOSH. PAEDIATRIC & ADOLESCENT CHRONIC FATIGUE SERVICES - UK 2015 [Available from: https://media.gosh.nhs.uk/documents/Specialist_Paediatric_CFSME_Services_England_updated_May_2015.pdf.

22. BACME. Specialist ME/CFS services map: British Association of Clinicians in ME/CFS; 2018 [Available from: https://bacme.info/services-map/.

23. NICE. Chronic fatigue syndrome/myalgic encephalomyelitis (or encephalopathy): diagnosis and management of chronic fatigue syndrome/myalgic encephalomyelitis (or encephalopathy) in adults and children. In: Turnbull N, Shaw EJ, Baker R, Dunsdon S, Costin N, Britton G, et al., editors. 2007.

24. Pendergrast T, Brown A, Sunnquist M, Jantke R, Newton JL, Strand EB, et al. Housebound versus nonhousebound patients with myalgic encephalomyelitis and chronic fatigue syndrome. Chronic illness. 2016;12(4):292-307.

25. Collard SS, Murphy J. Management of chronic fatigue syndrome/myalgic encephalomyelitis in a pediatric population: A scoping review. Journal of child health care : for professionals working with children in the hospital and community. 2020;24(3):411-31.

26. Gordon BA, Knapman LM, Lubitz L. Graduated exercise training and progressive resistance training in adolescents with chronic fatigue syndrome: a randomized controlled pilot study. Clinical rehabilitation. 2010;24(12):1072-9.

27. Clark LV, Pesola F, Thomas JM, Vergara-Williamson M, Beynon M, White PD. Guided graded exercise self-help plus specialist medical care versus specialist medical care alone for chronic fatigue syndrome (GETSET): a pragmatic randomised controlled trial. The Lancet. 2017;390(10092):363-73.

28. Al-Haggar M, Al-Naggar ZA, Abdel-Salam MA. Biofeedback and cognitive behavioral therapy for Egyptian adolescents suffering from chronic fatigue syndrome. Journal of pediatric neurology. 2015;04:161-9.

Confidential: For Review Only

29. Stulemeijer M, de Jong LW, Fiselier TJ, Hoogveld SW, Bleijenberg G. Cognitive behaviour therapy for adolescents with chronic fatigue syndrome: randomised controlled trial. BMJ (Clinical research ed). 2005;330(7481):14.

30. White PD, Goldsmith KA, Johnson AL, Potts L, Walwyn R, DeCesare JC, et al. Comparison of adaptive pacing therapy, cognitive behaviour therapy, graded exercise therapy, and specialist medical care for chronic fatigue syndrome (PACE): a randomised trial. Lancet. 2011;377(9768):823-36.

31. Brigden A, Beasant L, Gaunt D, Hollingworth W, Mills N, Solomon-Moore E, et al. Results of the feasibility phase of the managed activity graded exercise in teenagers and pre-adolescents (MAGENTA) randomised controlled trial of treatments for chronic fatigue syndrome/myalgic encephalomyelitis. Pilot and Feasibility Studies. 2019;5(1):151.

32. Castro-Marrero J, Sáez-Francàs N, Santillo D, Alegre J. Treatment and management of chronic fatigue syndrome/myalgic encephalomyelitis: all roads lead to Rome. Br J Pharmacol. 2017;174(5):345-69.

33. Kingdon C, Giotas D, Nacul L, Lacerda E. Health Care Responsibility and Compassion-Visiting the Housebound Patient Severely Affected by ME/CFS. Healthcare (Basel, Switzerland). 2020;8(3).

Confidential: For Review Only
