# Supplementary material for: Management of severe ME/CFS in children and young people in the UK: a British Paediatric Surveillance Unit study
Source: BMJ Paediatr Open. 2024 Mar 7;8(1):e002436. doi: 10.1136/bmjpo-2023-002436 (PMC10921487; doi:10.1136/bmjpo-2023-002436)
Supplement: Reviewer comments [file bmjpo-2023-002436.reviewer_comments.pdf]

PEER REVIEW HISTORY

BMJ Paediatrics Open publishes all reviews undertaken for accepted manuscripts. Reviewers are asked to complete a checklist review form and are provided with free text boxes to elaborate on their assessment. These free text comments are reproduced below.

ARTICLE DETAILS

**TITLE (PROVISIONAL)**	Management of Severe ME/CFS in Children and Young People in the UK: a British Paediatric Surveillance Unit study
**AUTHORS**	Royston, Alexander Peter<br>Burge, Sarah<br>Idini, Ilaria<br>Brigden, Amberly<br>Pike, Katharine Claire

VERSION 1 – REVIEW

**REVIEWER**	Dr. david Vickers<br>ccs nhs rust, paediatrics
**REVIEW RETURNED**	05-Jan-2024

**GENERAL COMMENTS**	This study is useful in that it identifies issues with management of these CYP, including lack of adherence to NICE guidance and inconsistent provision of support across the country.

VERSION 1 – AUTHOR RESPONSE

Reviewer: 1
Dr. david vickers, ccs nhs rust
Comments to the Author
This study is useful in that it identifies issues with management of these CYP, including lack of adherence to NICE guidance and inconsistent provision of support across the country.

Author RESPONSE:
Dear Dr Vickers, thank you for your kind comments. We agree that this is important research and are glad that you find it useful.
